# The role of SARS-CoV-2 aerosol transmission during the COVID-19 pandemic

**DOI:** 10.1098/rsfs.2022.0003

**Published:** 2022-02-11

**Authors:** Julian W. Tang, Linsey C. Marr, Yuguo Li, Ian Eames

**Affiliations:** ^1^ Respiratory Sciences, University of Leicester, Leicester, UK; ^2^ Civil and Environmental Engineering, Virginia Tech, VA, USA; ^3^ Department of Mechanical Engineering, The University of Hong Kong, Hong Kong Special Administrative Region, People's Republic of China; ^4^ Centre for Engineering in Extreme Environments, University College London, Gower Street, London WC1E 7JE, UK

**Keywords:** role, SARS, CoV-2, aerosol, transmission, COVID-19

## Abstract

The COVID-19 pandemic, caused by the virus SARS-CoV-2, has touched most parts of the world and devastated the lives of many. The high transmissibility coupled with the initial poor outcome for the elderly led to crushingly high fatalities. The scientific response to the pandemic has been formidable, aided by advancements in virology, computing, data analysis, instrumentation, diagnostics, engineering and infection control. This has led to improvements in understanding and has helped to challenge some established orthodoxies. Sufficient time has elapsed since the start of the COVID-19 pandemic that a clearer view has emerged about transmission and infection risks, public health responses and related societal and economic impacts. This timely volume has provided an opportunity for the science community to report on these new developments.

Tang *et al*. [1] set the scene for this volume with a comparative cultural and political analysis on how countries initially responded to the pandemic, to explain the successful initial response of Australasia and East/Southeast Asia compared to Europe and North America and their corresponding COVID-19 case numbers and fatalities. The paper describes the types of early actions taken by these different populations, as led by their respective governments; these are important lessons for the future.

The debate about how SARS-CoV-2 was mostly transmitted (by aerosol, droplet or contact) was one of the dominating issues during the early pandemic, as it affected the type of personal protective equipment (masks and face coverings) that healthcare workers and later on, the public, would be recommended to wear. It also impacted the larger scale public health measures that were to be implemented, such as social distancing (at least 1–2 m between people indoors) and ventilation, with the related fallout on businesses (e.g. hospitality and travel) and the overall economy. In the absence of vaccines and antiviral drugs in the early pandemic period, the population's acceptance and compliance with these non-pharmaceutical interventions were key in reducing the spread of the virus in every country. Although diagnostic testing data were sparse and patchy, globally, during the early pandemic, it quickly emerged that populations that reacted promptly by imposing universal masking, along with early border closures, and a rapid expansion of testing with enforced isolation for those infected and quarantine for their contacts, had the lowest COVID-19 case numbers and deaths.

The rest of this Special Issue focuses on this specific aspect of the aerosol transmission of SARS-CoV-2.

Tellier [2] starts by reviewing the accumulating evidence to support this, as obtained from a variety of sources, including controlled laboratory and animal studies, detection of aerosolized viruses in exhaled breath and in air samples collected indoors, as well as a review of real-world outbreak investigations. Perhaps most importantly, he argues that the very nature of human exhalation flows from a virus laden oro- and nasopharyngeal cavity—will generate virus aerosols—and that this mechanism is not just limited to coronaviruses, but also influenza, for which there is also a large body of evidence supporting aerosol transmission.

Eames & Flor [3] examine in more detail the dynamics of human exhaled airflows and how these can carry the virus through the air to potentially infect others. In particular, they analyse how near-source turbulent flows and thermal buoyancy effects interact with ambient air flows, temperature and relative humidity to cause far-field spreading effects, and how these can then interact with room ventilation. Understanding the nature of these complex flows in indoor spaces, where most viral transmission occurs, is crucial in optimizing how ventilation can reduce airborne virus spread.

Archer *et al*. [4] compared the numbers and mass concentrations of particles produced by adults and children when breathing, talking and singing. They found that the numbers and masses of particles produced are similar in children and adults, with greater differences seen between the different modalities, breathing ≪ talking < singing, all of which produce more particles if performed more loudly. Along with several studies showing that SARS-CoV-2-infected children produce just as much virus for just as long as adults [5–7], it is now understood and accepted that children can be just as potent a source of the virus, despite many of them being less symptomatic than adults. Indeed, with the much higher contact rates of children in schools, we have seen large outbreaks in schools throughout the COVID-19 pandemic, prior to the widespread rollout of the COVID-19 vaccine to children [8–10].

Real-world COVID-19 outbreaks are also described. Feathers *et al*. [11] investigated a SARS-CoV-2 outbreak among their staff and patients in a hospice, where the demise of already terminally ill patients was likely hastened by COVID-19. This outbreak was eventually terminated with the use of enhanced aerosol infection control measures, including the universal surgical masking for all staff while on hospice grounds, increased ventilation (by opening all windows to some degree) and the introduction of social distancing in communal areas.

Wang *et al*. [12] described an outbreak that involved 34 residents (an attack rate of 25.4%) in a Hong Kong high-density housing estate. These old estates consisted of multiple self-contained flats of 6–10 m^2^ or less, each with their own bathrooms. Such complex plumbing (including some *ad hoc* user modifications) was leaky in places, and in such overcrowded conditions could have been a source of airborne virus. Toilet flushing and the use of extractor fans, in the presence of dried U-traps, generated ambient airflows via chimney and stack aerosol effects that could carry the virus through multiple units. Although it was difficult to prove that all cases were transmitted by aerosols, viral sequencing of 17 out of 23 cases demonstrated virtually identical viral sequences, indicating a likely common source. High-density living conditions are always a risk for the person-to-person spread of transmissible pathogens. The outbreak of COVID-19 in Singaporean migrant workers is another example of this [13].

Finally, Henriques *et al*. [14] integrate some of these aerosol transmission concepts into a SARS-CoV-2 transmission and exposure risk model for finite-volume indoor air spaces, including the effects of various interventions, including masking, vaccination (or natural immunity), ventilation and social distancing. Using contemporaneous data from multiple studies to accurately parameterize the model, the ultimate goal is for users to input their specific parameters into the model to then obtain a risk assessment of SARS-CoV-2 transmission and infection in their workspaces. Although this model only explores the risk due to the presence of well-mixed virus in finite-volume indoor air spaces, shorter range, more intense ‘conversational’ exposure modelling is being currently developed.

With any new emerging pathogen, there will be initially some controversies and debates about the way they are transmitted, for example, with HIV and the famous Florida dentist cluster [15]; Nipah virus, which only appeared to infect Chinese or Indian but not Muslim Malay abbatoir workers [16]; SARS-CoV-1 that seemed to disproportionately infect healthcare workers [17]; MERS-CoV [18] and avian influenzas [19], which do not appear to transmit efficiently between humans; and ongoing debates about whether Ebola can spread via aerosols [20]. Understanding the main route of transmission of any pathogen is critical to developing effective interventions to limit its spread. Even an incomplete understanding is helpful, as interventions can be developed to cover multiple potential transmission routes—as long as an early, precautionary approach is adopted.

This Special Issue illustrates the importance and the role of aerosol transmission for SARS-CoV-2, including some of the underlying reasons why there was such resistance to this concept during the critical early phase of the pandemic.

## Data accessibility

This article has no additional data.
